# Effect of RF and Anti-CCP antibody for Rheumatoid Arthritis diagnosis: A comparative study

**DOI:** 10.6026/973206300214420

**Published:** 2025-12-15

**Authors:** Rohit Dubepuria, Dinesh Singh Mahor, Shubham Gupta, Nishtha Jain

**Affiliations:** 1 Department of General Medicine, Chirayu Medical College & Hospital, Bhopal, Madhya Pradesh, India; 2 Department of General Medicine, Government Medical College, Sheopur, Madhya Pradesh, India; 3 Department of General Medicine, R.D Gardi Medical College, Ujjain, Madhya Pradesh, India

**Keywords:** Rheumatoid arthritis, Anti-CCP antibodies, rheumatoid factor, diagnostic accuracy, autoimmune disease

## Abstract

Rheumatoid arthritis (RA) is a long-term autoimmune disease that needs to be diagnosed as soon as possible in order to achieve maximum treatment results. Although rheumatoid factor (RF) has been a conventional serologic test, the anti-cyclic citrullinated peptide (anti-CCP) antibodies are now considered a more specific diagnostic test. Therefore, it is of interest to determine the diagnostics performance of RF and anti-CCP antibody tests in suspected RA patients in a tertiary care hospital. One hundred and eighty participants will be recruited, that is, 120 participants diagnosed with RA clinically (according to the 2010 ACR/EULAR definitions) and 60 age-matched controls. RF in serum samples was studied by nephelometry and the anti-CCP antibodies through enzyme-linked immunosorbent assay (ELISA). Anti-CCP antibodies were more specific (96.7% vs. RF, 78.3%), whereas the sensitivity was also similar (81.7% vs. 75.0% respectively). Anti-CCP (96.1) had a better positive predictive value than RF (85.7%). The sensitivity (88.3%) and specificity (95.0%) with combined testing were improved. Anti-CCP was found to have significant correlations with disease activities and radiological erosions. Data shows uphold anti-CCP antibodies as a better diagnostic tool than RF in Diagnosing RA and the combination of the two tests provides the best diagnostic value in the early stages of the disease and prognostication.

## Background:

Rheumatoid arthritis (RA) is an autoimmune disorder systemic and that is chronic inflammatory polyarthritis and it is prevalent in around 0.5-1.0 percent of the world population [1]. Synovial joints are the major targets of the disease, which causes progressive cartilage and bone degeneration, which eventually cause a significant functional disability and low quality of life [2]. It is essential to diagnose and treat disease-modifying antirheumatic drugs (DMARDs) early so as to prevent the irreversible joint damage and enhance long-term outcomes [3]. Serological biomarkers are important in the diagnosis and classification of RA. Rheumatoid factor (RF) is a type of autoantibody that is used to diagnose a disease that acts against the Fc sub-unit of immunoglobulin G and was first used more than 60 years ago [4]. Nevertheless, RF does not show much specificity because it may be observed in other autoimmune diseases, persistent infections and even in some healthy people, especially in the elderly groups [5]. This generalization usually causes diagnostic ambiguity and delayed therapeutic intervention. The identification of anti-cyclic citrullinated peptide (anti-CCP) antibodies has changed the serological diagnosis of RA [6]. The antibodies are specific to citrullinated proteins resulting as a consequence of post-translational modification of arginine residues by peptidylarginine deiminase enzymes [7]. Various studies have shown that anti-CCP antibodies have high specificity of RA than RF with a score of 90-98% [8]. In addition, the anti-CCP antibodies can be identified many years before the disease progresses to clinical manifestations with a possible prospect of the prevention and early diagnosis [9]. Recent studies have conducted a comparison of diagnostic value of these serological markers in different populations. The meta-analysis conducted by Nishimura *et al.* showed that anti-CCP antibodies were more specific (96) as compared to RF (85) in the diagnosis of RA [10]. On the same note, the same has been reported by studies that have been conducted on Asian populations, with the sensitivities of the two markers varying, indicating some ethnic and genetic effects on the antibody expression pattern [11]. Although there is an increasing body of evidence, scanty data on Indian tertiary care settings on the performance of these markers against each other in daily practice exists. Clinical significance is not limited to diagnosis because RF and anti-CCP antibodies can be used prognostically. The positivity of the anti-CCP has been linked with aggressive disease phenotypes, rapid radiological progression and with high probability of extra-articular manifestation [12]. Therefore, it is of interest to compare the diagnostic capabilities of RF and anti-CCP antibody tests in patients with clinically suspected rheumatoid arthritis in a tertiary care hospital, assessing the sensitivity and specificity of these tests, as well as their predictive value and the correlation with the disease activity and radiological severity.

## Materials and Methods:

## Study design and setting:

This was a cross-sectional comparative study that took place within the Department of Medicine and in cooperation with the Department of Biochemistry and Rheumatology in a teaching hospital of the tertiary care in 18 months (January 2023 to June 2024).

## Sample size and study population:

The total number of subjects recruited to the study was 180, which included 120 patients who had clinically diagnosed rheumatoid arthritis and 60 age and sex-matched healthy controls. The sample size involved was calculated at a sensitivity of 75 percent as expected with 95 percent confidence interval and 5 percent margin of error, which needed a minimum of 115 patients with RA.

## Inclusion criteria:

[1] In the case of RA group: (1) 18-75 years old, (2) RA diagnosis based on 2010 American College of Rheumatology/European League Against Rheumatism (ACR/EULAR) classification criteria, (3) a disease of the less than 5-year duration and (4) patient willingness to take part in the study.

[2] In the case of the control group: (1) Age- and sex-matched healthy subjects, (2) no autoimmune disorders history, (3) no chronic inflammatory diseases and (4) no family history of rheumatoid arthritis.

## Exclusion criteria:

Patients were excluded in the event that they had: (1) other autoimmune diseases (systemic lupus erythematosus, Sjogren syndrome and scleroderma), (2) chronic liver disease or hepatitis B/C infection, (3) chronic kidney disease (eGFR <30 mL/min/1.73m 2), (4) active malignancy, (5) other infections in the past 4 weeks, or (6) pregnancy or lactation.

## Clinical assessment:

Clinical assessment which incorporated thorough history, physical and joint examination of all the patients with RA took place. The Disease Activity Score-28 (DAS28) was used to determine the disease activity together with erythrocyte sedimentation rate (ESR). The Health Assessment Questionnaire-Disability Index was used to assess functional status (HAQ-DI). Hands and feet radiological evaluation was conducted and erosive changes were evaluated based on the modified Sharp-van der Heijde score.

## Laboratory methods:

All the participants were sampled by availing fasting venous blood samples (5 mL) under aseptic conditions. The serum was centrifuged at 3000 rpm (10min) and then frozen at -20o C pending analysis. Rheumatoid Factor (RF) Testing: The RF was detected with the help of the particle-enhanced immunoturbidimetric assay on a nephelometer (BN ProSpec System, Siemens Healthcare Diagnostics). The values above 20 IU/mL were taken as positive according to the specifications of the manufacturers and the institutional values.

## Anti-CCP antibody testing:

Anti-CCP antibodies (second-generation) were measured with the help of enzyme-linked immunosorbent assay (ELISA) kit (QUANTA Lite CCP3 IgG ELISA, Inova Diagnostics, USA). The cutoff value of positive results was more than 20 U/mL as the manufacturer specified. Each of the tests was done in duplicate and the quality control measurements carried out with internal quality control sera. The clinical diagnosis was unknown to both technicians who conducted the assays and interpreted the results.

## Statistical analysis:

The analysis of the data was conducted with the SPSS version 25.0 (IBM Corp., Armonk, NY, USA). Continuous variables as mean ± standard deviation (SD) were compared between independent t-test or Mann-Whitney U test according to the analysis of data distribution that was determined by the use of Shapiro-Wilk test. Categorical variables were given in terms of frequencies and percentages and their analysis was performed by chi-square test or exact test. The sensitivity, the specificity, positive predictive value (PPV), the negative predictive value (NPV) and the accuracy of the diagnosis were determined using 95 percent confidence intervals. The level of antibodies and the parameters of the disease were correlated with each other with the help of Pearson or Spearman correlation coefficient. With a p-value of less than 0.05, the p-value was found to be statistically significant.

## Results:

The study included 120 RA patients and 60 healthy controls. The mean age of RA patients was 48.6 ± 12.4 years, with female predominance (74.2%). The control group had a mean age of 47.8 ± 11.9 years with 73.3% females, showing no significant difference (p>0.05). The mean disease duration in RA patients was 2.4 ± 1.6 years. The mean DAS28-ESR score was 4.8 ± 1.3, indicating moderate to high disease activity in the majority of patients. Clinical manifestations included symmetric polyarthritis (91.7%), morning stiffness >1 hour (85.8%) and radiological erosions (56.7%). Table 1 (see PDF) presents the distribution of RF and anti-CCP antibody positivity among study groups. Among RA patients, anti-CCP antibodies were positive in 98 patients (81.7%), while RF was positive in 90 patients (75.0%). In the control group, 2 individuals (3.3%) tested positive for anti-CCP antibodies, whereas 13 (21.7%) were RF positive. The difference in specificity between the two markers was statistically significant (p<0.001). Table 2 (see PDF) demonstrates the comparative diagnostic performance parameters of RF and anti-CCP antibodies. Anti-CCP antibodies showed superior specificity (96.7%) compared to RF (78.3%, p<0.001), while maintaining comparable sensitivity (81.7% vs. 75.0%, p=0.18). The positive predictive value was significantly higher for anti-CCP (96.1%) than RF (85.7%, p=0.008). Combined testing (either RF or anti-CCP positive) enhanced sensitivity to 88.3% while maintaining excellent specificity of 95.0%. Table 3 (see PDF) shows the correlation between serological markers and disease activity parameters. Anti-CCP antibody levels demonstrated significant positive correlation with DAS28-ESR scores (r=0.412, p<0.001), ESR (r=0.386, p<0.001), C-reactive protein (r=0.394, p<0.001) and radiological erosion scores (r=0.448, p<0.001). RF levels showed moderate correlation with DAS28-ESR (r=0.298, p=0.001) and weaker correlation with radiological changes (r=0.276, p=0.002). Patients positive for both RF and anti-CCP antibodies demonstrated significantly higher disease activity (mean DAS28-ESR: 5.2 ± 1.2) compared to those positive for only one marker (4.3 ± 1.3, p=0.003) or seronegative patients (3.8 ± 1.1, p<0.001). Radiological erosions were present in 65.9% of dual-positive patients compared to 43.8% in single-positive and 25.0% in seronegative patients (p<0.001). The mean anti-CCP antibody level in RA patients was 186.4 ± 124.7 U/mL compared to 8.6 ± 6.2 U/mL in controls (p<0.001). Similarly, mean RF levels were significantly elevated in RA patients (112.8 ± 98.6 IU/mL) versus controls (14.2 ± 8.4 IU/mL, p<0.001). Among RA patients with erosive disease, mean anti-CCP levels were significantly higher (218.6 ± 132.4 U/mL) than in those without erosions (146.2 ± 108.3 U/mL, p=0.001).

## Discussion:

The current research offers a detailed comparative statistics of diagnostic effectiveness of RF and anti-CCP antibodies tests in tertiary care hospital population which indicates a high specificity of anti-CCP antibodies and also, it shows the additive diagnostic value of combined testing. Our results are consistent with the paradigm shift in RA diagnostics that is currently going on and focuses on high specificity of serological indicators in the early and accurate detection of the disease. The sensitivity of the anti-CCP antibodies (81.7) in our study is in line with international literature that has sensitivities of 68-82% [13]. The slightly increased sensitivity over RF (75.0%) is an indication that anti-CCP antibodies identify a wider range of RA patients such as those who might be RF-negative. This is especially important considering that 10-30% of RA patients are seronegative to RF and these results in delays in diagnosis over and again [14]. As our data showed, anti-CCP positive and RF negative patients were 13.3 per cent of the total RA patients, which highlights the quality of anti-CCP testing in this population. The most remarkable result was the significantly high specificity of anti-CCP antibodies (96.7) in comparison with RF (78.3). This 18.4 percentage difference in specificity has significant clinical implications, causing false-positive diagnoses to be significantly decreased, as well as avoiding therapeutic interventions and anxiety of the patient. The strong rate of false-positive of RF in our control group (21.7) is because it is well documented among older people, chronic infections and other autoimmune diseases [15]. Conversely, a low percentage of 3.3% of controls were positive in anti-CCP antibodies which validated its specificity to the disease. These results are supported by the recent research indicating the better discriminatory effect of anti-CCP. Aggarwal *et al.* systematic review showed that the anti-CCP pooled specificity was 96.6% compared to RF pooled specificity of 85.1, which is quite similar to our findings [6]. In the same manner, van Venrooij *et al.* underscored the fact that anti-CCP antibodies are rare in healthy people or non-RA inflammatory states hence extremely useful in confirming a diagnosis [7]. Positive predictive values of the anti-CCP antibodies (96.1) were higher than RF (85.7), which means that a positive result on the anti-CCP test is more likely to be accurate when it comes to the diagnosis of RA. This is especially applicable in the clinical setting where there is a difficulty in differentiating RA and other inflammatory arthritides. Although the negative predictive value is low with both markers, they indicate that seronegativity does not rule out RA which is why clinical criteria are very important in diagnosis. There was increased diagnostic accuracy with a combined testing strategy and dual testing (either positive) demonstrated 88.3% sensitivity and 95.0% specificity. This method helps to capture RF-positive/anti-CCP-negative and anti-CCP-positive/RF-negative subgroups, which is the best to be able to reach maximum case detection. The positive result of both of these markers, however, to get nearly perfect specificity (98.3%) had a substantial negative impact on sensitivity (68.3%), which could miss up to one-third of RA patients. Thus, the sequential approach of testing, the first stage of which is anti-CCP screening and RF testing in case of the negative result, could be the most cost-effective diagnostic algorithm [8].

Correlation analysis showed that anti-CCP antibody levels had more associations with disease activity markers, functional disability and radiological damage in relation to RF. The high correlation between the anti-CCP levels and the erosion scores (r=0.448, p<0.001) confirms this point has been reported before where the anti-CCP antibodies have been suggested to be predictive biomarkers of the extreme, erosive disease phenotypes [9]. This prognostic dimension is the extension of anti-CCP testing use to beyond diagnosis to risk stratification and treatment planning. The profile of the most aggressive disease was observed in patients who were not only RF-positive but anti-CCP-positive as well, with better scores in DAS28 and an increased erosive load. This finding confirms existing data where dual seropositivity refers to a sub-group in need of a high-intensity early intervention with a combination of DMARDs or biologic therapy to avoid permanent joint destruction [10]. On the other hand, seronegative patients still exhibited less severe disease activity, although some of them also had erosions, which also demonstrates the heterogeneity of the disease. The strengths of our study involve rigorous research design with blinded laboratory tests, standardized clinical tests on validated measures (DAS28, HAQ-DI) and adequate sample size and controls. Radiological correlation is also included and this offers a good understanding of the prognostic value of these biomarkers. Nevertheless, there are a number of constraints, which should be considered. The cross-sectional design does not allow the determination of antibody performance in very early or undifferentiated arthritis, where diagnostic performance might not be the same. Additional information would be given by longitudinal studies of seroconversion and disease progression. Moreover, our population of study was the established RA patients during 5 years of diagnosis; the pre-clinical or very early disease must be examined separately. Another practical factor that we have not examined in our study formally is that of costs. Although anti-CCP may tend to be more costly than RF in resource-constrained environments, higher specificity could be worth the cost as it may prevent wrongful diagnosis and incorrect medication. Policy decisions would be made based on cost-effectiveness analyses using diagnostic accuracy, therapeutic implications and long-term outcomes [11]. New studies on other citrullinated peptide antibodies and other autoantibody systems (anti-carbamylated protein antibodies, anti-PAD4 antibodies) can further optimize the RA diagnostics [12, 13]. Combination of serological with genetic risk factors (HLA-DRB1 shared epitope), imaging (musculoskeletal ultrasound, MRI) and molecular (molecular signatures) biomarkers is the future of precision medicine in rheumatology. Multiple biomarker panels with a combination of more than one antibody specificity could increase sensitivity and specificity over the current single-marker methods [14, 15]. Our clinical consequences are indicative of the adoption of anti-CCP antibody test as an initial serological study in suspected patients with RA with RF being a complementary and not the primary serological examination [14]. Revised diagnostic algorithms must place more emphasis on anti-CCP testing especially in the cases of ambiguous presentation or high diagnostic specificity is needed. Nevertheless, the fact that seronegative RA cannot be eliminated shows that no serological test can substitute a thorough clinical evaluation, which is conducted on the basis of current classification criteria [16, 17].

## Conclusion:

Anti-CCP antibodies have a high diagnostic specificity and predictive quality than RF in the diagnosis of rheumatoid arthritis and have similar sensitivity. Combination tests make the diagnosis more accurate especially where either of the markers positive is the criterion. Anti-CCP antibodies also show better correlations with disease severity and radiological progression, which are independent of diagnosis and warrant their place in diagnostic establishment as well as prognostic determination in tertiary care contexts.

## References

[1] Smolen S (2016). Lancet..

[2] Scott D.L (2010). Lancet..

[3] van der Woude D (2018). Best Pract Res Clin Rheumatol..

[4] https://healthcare-bulletin.co.uk/article/volume-14-issue-1-pages458-462-ra/.

[5] Hashiam MD (2022). Int J Health Sci..

[6] Aggarwal R (2009). Arthritis Rheum..

[7] van Venrooij W.J (2011). Nat Rev Rheumatol..

[8] Whiting P.F (2010). Ann Intern Med..

[9] https://onlinejima.com/journals_doc/download-journals/2021/july/35-37.pdf.

[10] Nishimura K (2007). Ann Intern Med..

[11] Wang F (2021). PLoS One..

[12] Waqas M (2024). Pakistan Congress of Zoology..

[13] Taylor P (2011). Autoimmune Dis..

[14] Aletaha D (2013). Ann Rheum Dis..

[15] Ingegnoli F (2013). Dis Markers..

[16] Dedwal A.K (2017). IJMMTD..

[17] Hammam N (2024). Rheumatol Immunol Res..

